# ω-3 and ω-6 Polyunsaturated Fatty Acids Regulate the Proliferation, Invasion and Angiogenesis of Gastric Cancer Through COX/PGE Signaling Pathway

**DOI:** 10.3389/fonc.2022.802009

**Published:** 2022-02-17

**Authors:** Jiachi Ma, Chensong Zhang, Wanqing Liang, Lei Li, Jun Du, Chengwu Pan, Bangling Chen, Yuzhong Chen, Yuanpeng Wang

**Affiliations:** ^1^ Department of Oncological Surgery, The First Affiliated Hospital of Bengbu Medical College, Bengbu, China; ^2^ Department of General Surgery, The Second Hospital of Bengbu Medical College, Bengbu, China

**Keywords:** ω-3 PUFAs, ω-6 PUFAs, angiogenesis, metastasis, gastric cancer

## Abstract

**Background:**

This study aims to investigate the effects of ω-3, ω-6 polyunsaturated fatty acids (PUFAs), and their middle metabolites prostaglandin (PGE)2 and PGE3 on proliferation, invasion, and angiogenesis formation of gastric cancer cells and to explore associated mechanism.

**Methods:**

RT-PCR and ELISA were used to detect the expression of cyclooxygenase (COX)-1 and COX-2 in gastric cancer cell lines. The effect of ω-3, ω-6, PGE2, and PGE3 on the proliferation, invasion, and angiogenesis of gastric cancer cells were measured by cell proliferation, invasion, and angiogenesis assay *in vitro*. COX-2 small interfering RNA (siRNA) was transfected into gastric cancer cells, and the expression of COX-2 protein was detected by Western blot. COX-2 gene silencing influencing proliferation, invasion, and angiogenesis potential of gastric cancer cells was detected by WST-1, transwell chamber, and angiogenesis assay, respectively.

**Results:**

COX-2 was only expressed in MKN74 and MKN45 cells. In gastric cancer cell lines with positive COX-2 expression, ω-6 and PGE2 could significantly enhance the proliferation, invasion, and angiogenesis of gastric cancer cells, and after transfection with COX-2 siRNA, the effects of ω-6 and PGE2 on enhancing the proliferation, invasion, and angiogenesis of gastric cancer cells were significantly attenuated; ω-3 and PEG3 could inhibit the proliferation, invasion, and angiogenesis of gastric cancer cells. In gastric cancer cell lines with negative COX-2 expression, ω-6 and PGE2 had no significant effect on the proliferation, invasion, and angiogenesis of gastric cancer; ω-3 and PGE3 could significantly inhibit the proliferation, invasion, and angiogenesis of gastric cancer.

**Conclusion:**

ω-6 PUFAs reinforce the metastatic potential of gastric cancer cells *via* COX-2/PGE2; ω-3 PUFAs inhibit the metastatic potential of gastric cancer *via* COX-1/PGE3 signaling axis.

## Introduction

Gastric cancer is one of the common malignant tumors, with an incidence of 17.6/100,000 worldwide, about 1.1 million new cases per year, accounting for 5.6% of all new cases of malignant tumors and ranking fifth, and it also ranks fourth due to 770,000 deaths it causes (1). The main cause of death in patients with gastric cancer is metastasis; the liver is the most common hematogenous metastatic organ of gastric cancer, and the incidence of liver metastasis in gastric cancer ranges from 17% to 29% (2, 3). Patients with liver metastasis from gastric cancer have a very poor prognosis and a 5-year survival rate of less than 10% (4). Although there are many studies on liver metastasis of gastric cancer, the molecular mechanism of liver metastasis of gastric cancer has not been elucidated so far, and there is no effective treatment in clinical practice. Therefore, it is important to deeply study the mechanism of liver metastasis of gastric cancer and take targeted interventions to improve the survival rate and quality of life of patients with gastric cancer.

Polyunsaturated fatty acids (PUFAs) ω-3 and ω-6 are the main components of cell membrane structure, which also are essential fatty acids for human body. Recent studies have shown that ω-6 PUFAs can promote the occurrence, progression, and metastasis of malignant tumors, while ω-3 PUFAs has anticancer effects. ω-3 and ω-6 PUFAs play an important role in remodeling the microenvironment to regulate tumor metastasis, but the regulated mechanism is still unclear (5). PUFAs are a class of fatty acids containing double bonds on the carbon chain (6), which, in addition to providing energy for the body, are also involved in the composition of cell membrane lipids and are important substances in the regulation of cellular metabolism and cell signaling. PUFAs are classified into ω-3 system, ω-6 system, ω-7 system, and ω-9 system according to the position of the first double bond in the carbon chain as counted from the methyl end (7). Among them, ω-3 and ω-6 PUFAs are the most common PUFAs. The ω-3 and ω-6 PUFAs are the main components of various biofilm structures, which play an indispensable role in maintaining the normal physiological metabolism of the human body and are essential fatty acids in the human body. The ω-3 PUFAs have good immunomodulatory effects and can inhibit local chronic inflammatory responses by regulating the cell microenvironment, stabilizing cell membranes, and regulating cell proliferation and differentiation, which in turn play a role in the prevention and treatment of tumors (8–10). The applicant’s previous studies have shown that, PUFAs can affect the invasion, proliferation, and angiogenesis of gastric cancer cells, and the role of PUFAs is closely related to their metabolites prostaglandin (PGE) and cyclooxygenase (COX) on the nuclear membrane of tumor cells *in vivo*, while the expression of PGE and COX is closely related to lymphatic metastasis of gastric cancer (11). The ω-3 and ω-6 PUFAs, as essential fatty acids in the human body, can play a role in inhibiting tumor invasion by reducing ω-6 PUFAs containing foods and appropriately increasing ω-3 PUFAs containing foods in the daily diet (12). COX is an essential enzyme for the synthesis of prostaglandin (PG) and a key rate-limiting enzyme in the initial step of PG synthesis. Cyclooxygenase has two isozymes, COX-1 and COX-2. COX-1 is a structural enzyme that is expressed in most normal tissues, and COX-1 promotes prostaglandin production, thereby maintaining normal human function (13); COX-2 is an inducible enzyme that is rarely expressed in normal tissues, but often highly expressed in tumor cells, such as melanoma, colon cancer, breast cancer, liver cancer, cervical cancer, esophageal cancer, pancreatic cancer, and gastric cancer (14). At present, studies have confirmed that dietary polyunsaturated fatty acid is closely related to the occurrence and metastasis of gastric cancer (15). Among them, ω-6 PUFAs (arachidonic acid) can bind to cyclooxygenase 2 (COX-2) to produce PGE2 and enhance cancer cell invasion; ω-3 PUFAs (eicosapentaenoic acid) can bind to cyclooxygenase 1 (COX-1) to produce PGE3 and inhibit the activity of COX-2, reduce the production of PGE2, and inhibit cancer cell invasion (16).

Currently, there are few studies on the role of ω-3 PUFAs and ω-6 PUFAs in gastric cancer metastasis and their mechanisms. However, the antitumor effect of ω-3 PUFAs and the tumor-promoting effect of ω-6 PUFAs are complex processes involving multiple factors and multiple levels and are interrelated, and there are still many issues to be elucidated. For this reason, this study focused on exploring the mechanism of ω-3 PUFAs, ω-6 PUFAs, and their intermediate metabolites PGE2 and PGE3 on gastric cancer progression and metastasis, elucidating the biological characteristics and mechanism of PUFAs affecting gastric cancer metastasis, exploring the molecular targets affecting gastric cancer metastasis, and providing a theoretical basis and new way for the clinical application of ω-3 and ω-6 PUFAs in the prevention and treatment of gastric cancer.

## Materials and Methods

### Cell Lines and Culture

The cell lines derived from human gastric carcinoma were examined: MKN45, MKN74, and NUGC-4 cell lines were obtained from Japanese Riken Cell Bank (Tsukuba, Japan). All cell lines were maintained in RPMI 1640 (Sigma Chemical Co., St. Louis, MO, USA) added with 10% heat-inactivated fetal bovine serum (FBS). Human umbilical vein endothelial cells (HUVECs) were obtained from Kurabo Co. (Osaka, Japan). HUVECs were maintained in HuMedia-EG2 medium supplemented with 2% FBS, 5 ng/ml basic fibroblast growth factor, 10 μg/ml heparin, 10 ng/ml epidermal growth factor, and 1 μg/ml hydrocortisone according to the supplier’s instructions (Kurabo Co.). All cells were incubated at 37°C in a humidified atmosphere of 5% CO_2_ in air.

### RT-PCR Analysis of COX-1 and COX-2 mRNA Expression

Total RNA was extracted from gastric cancer cell lines by an Isogen Kit (Nippon Gene, Tokyo, Japan), and quantities were determined spectrophotometrically. The 1 μg of total RNA aliquots was reverse-transcribed into cDNA using the SuperScript III system (Invitrogen, San Diego, CA, USA) in a PCR Thermal Cycler (model TP3000; Perkin-Elmer, Norwalk, CT, USA). Reaction mixture aliquots (1 μl) were used as templates for PCR analysis. Amplification reactions were performed in a DNA Thermal Cycler.

The primer sequences and PCR conditions are shown in **Table 1**. The amplified DNA fragments were resolved by electrophoresis in 1.5% agarose gels containing ethidium bromide.

**Table 1:** Primer sequence and PCR condition.

Gene Name	Primer Sequences	Tm (°C)	Cycles	Length (bp)	Accession number
COX-1	F: 5’-CTGGAGGGTGGACTTGTCAT-3’	58	35	250	NM_001003023
	R: 5’-ACATTCTAGGTTGTCGGCCA-3’				
COX-2	F: 5’-GAGAGAAGGAAATGGCTGCG-3’	58	35	203	NM_001003354
	R: 5’-ACACACAGCCAGTCAACGAG-3’				

### Enzyme-Linked Immunosorbent Assay for COX-1 and COX-2 Protein Measurement

To determinate the COX-1 and COX-2 protein measurement, HUVECs and cells of the three gastric cancer cell lines (MKN74, MKN45, NUGC-4) were seeded at a density of 2 × 10^5^ cells/ml cells into 12-well plates and cultured overnight, following which the medium in each well was replaced and the cells cultured for a further 48 h. Cell numbers were determined, and the culture media were harvested and microfuged at 1,500 rpm for 15 min to remove the particles. The supernatant liquid were frozen at −80°C until used in ELISA assay. The concentration of COX-1 and COX-2 in supernatants of per 2 × 10^5^/ml cells was measured by ELISA kit (R&D Systems, Minneapolis, MN, USA) according to the manufacturer’s instructions.

### Design and Synthesis of siRNA and Transfection Into Gastric Cancer Cells

Two specific small interfering RNAs (siRNAs) were designed based on the coding region gene sequence of the human COX-2 gene, and the COX-2 siRNA sequences were 5′-GCCAAGGAGUGC UAAAGAA-3′ and 5′-CCAACACAGAAAUUGU-3′, and the control siRNA sequences were 5′-UUCUCCGAACGUGUCACGUTT-3′ and 5′-ACGUGACACGU CGGAGAATT-3′. After counting the two kinds of gastric cancer cells, they were seeded in cell culture dishes with a diameter of 35 mm at a density of 2 × 10^5^ cells/well and cultured overnight, followed by replacement with fresh culture medium containing 10% fetal bovine serum without antibiotics for another 24 h before transfection. A total of 500 μl of Opti-MEM^®^ I-reduced serum medium was used to dilute 200 nmol/L COX-2 siRNA or control siRNA, while 10 μl of LipofectAMINE™2000 was diluted with the same reagent. After standing at room temperature for 5 min, the two were quickly mixed and then allowed to stand at room temperature for 20 min. The mixture of siRNA-Lipofect AMINE™2000 (diluted by adding 1 ml culture medium) at a concentration of 100 pmol/L was then directly added to each cultured cell, followed by mixing well and placing in an incubator at 37°C for transfection. After 48 h of transfection, cells were collected for Western blotting assay to verify the silencing effect of COX-2 gene.

### Western Blot Was Used to Detect the Effect of COX-2 Gene Silencing on COX-2 Protein Expression in Gastric Cancer Cells

COX-2-expressing gastric cancer cells at 1 × 10^6^ cells/ml in the logarithmic growth phase were aspirated, the cells were lysed with a cell-lysis buffer, total protein was extracted and centrifuged at 500×*g* for 15 min at 4°C, and then the supernatant was collected to determine the protein concentration using the Bradford method. A total of 30 μg of sample protein was mixed well with an appropriate amount of solid-phase pH gradient strip solution, and electrophoresis was performed using a 10% SDS-PAGE gel for 2 h. The proteins on the gel plate after electrophoresis were transferred to PVDF membranes, followed by blocking with 5% skimmed milk powder for 2 h at room temperature and washing the membranes three times with TBST buffer. The membrane was immersed in blocking solution containing rabbit anti-human COX-2 monoclonal antibody (dilution ratio of 1:800), followed by reaction at room temperature for 2 h and the membrane washing three times with TBST solution; then the membranes were immersed in horseradish peroxidase-labeled goat anti-rabbit or anti-mouse IgG (dilution ratio of 1:2,000) solution, respectively, followed by placement overnight at 4°C, washing three times with TBST buffer, color development by ECL method, and scanning by computer. The gray value of the target band was determined using the image analysis software Image J. The relative expression level of the target protein was expressed as the ratio of the gray value of the target protein and the internal reference protein band, followed by plotting after statistical analysis.

### WST-1 Assay Examined the Effects of ω-3 PUFAs, ω-6 PUFAs, PGE2, and PGE3 on Proliferation of Gastric Cancer Cell

Gastric cancer cells expressing and not expressing COX-2 in the logarithmic growth phase were taken, and each group of cells were added to a 96-well culture plate at a density of 1 × 10^4^ cells/100 μl, respectively, with five replicating wells in each group, and the cells were cultured overnight to adhere and grow. The culture medium was replaced, and after another 72 h of culture, 100 μl CellTiter 96 aqueous solution reagent was added to each well and placed in a 37°C incubator for 4 h of reaction, and then the absorbance (*D* value) of the cells in each well at a wavelength of 490 nm was measured with a microplate reader to reflect the proliferation of the cells. The cell growth curve was plotted with time as the abscissa and the average D-ordinate.

### Transwell Chamber Assay Examined the Effects of ω-3 PUFAs, ω-6 PUFAs, PGE2, and PGE3 on Invasion of Gastric Cancer Cell

The *in vitro* invasion assay was performed using BioCoat Matrigel Invasion Chambers (Becton Dickinson, Bedford, MA, USA) according to the manufacturer’s instructions. Briefly, gastric cancer cells expressing and not expressing COX-2 in the logarithmic growth phase were used to adjust the single cell density to 2.0 × 10^5^cells/ml with different culture media containing 5% fetal bovine serum; the cells were seeded into transwell chambers with matrigel at the bottom, and the chambers were placed in 24-well cell culture plates, with 5 replicates for each group of cells. After 12 h of culture, chambers were removed, and cells that did not cross the membrane were wiped off with a cotton swab, rinsed three times with PBS, fixed in 4% formaldehyde solution for 5 min, and stained with Diff-Quick’s solution.

#### Result Interpretation

The number of penetrating cells within five fields was counted separately for each filter membrane under a light microscope (×100), and the average number of cells per field was calculated, in order to reflect the invasive ability of the cells.

### ω-3 PUFAs, ω-6 PUFAs, PGE2, and PGE3 Influence Angiogenesis *In Vitro*

HUVECs and human fibroblasts were seeded in a 24-well culture plate in a certain proportion and cultured together. On the second day, the culture medium was replaced and ω-3 PUFAs, ω-6 PUFAs, PGE2, and PGE3 at different concentrations were added, and then a transwell chamber with 0.45 μm microwells was placed in the 24-well culture plate. The bottom of the chamber was covered with polycarbonate membrane and transwell chambers with the wells contained 2 × 10^4^/ml gastric cancer cells to form a coculture system. The culture medium was changed every day, and after 11 days of coculture, the culture medium was removed and the culture wells were washed three times with a PBS solution and fixed with formaldehyde for 30 min, followed by vascular staining with CD31 antibody and then by natural drying. Angiogenesis in 10 different areas was photographed under a microscope and then each photograph was analyzed with vascular analysis software (Kurabo Co.); the total area or length of blood vessels in each photograph was calculated, and the standard amount of new blood vessels was expressed in pixels.

### Angiogenic Activity During Cocultivation With Gastric Cancer Cells and Regulation of Polyunsaturated Fatty Acid

To further investigate the effect of different gastric cancer cells on tubule formation by HUVECs. Transfected or nontransfected gastric cancer cells (MKN45 or NUGC-4) were cocultured with HUVECs and fibroblasts using a double chamber method in 24-well plates. MKN45 or NUGC-4 cells (2 × 10^4^ cells/ml) were planted in transwell chambers, consisting of polycarbonate membranes with 0.45 μm pores, and the cells adhere overnight. The transwell chambers were then placed in the HUVEC/fibroblast and coincubated in 24-well plates and the medium exchanged every 2 days. Cells were incubated for 12 days, and HUVEC tubule formation was determined as described above.

### Statistical Analysis

Statistical comparisons were made using Student’s *t*-test for paired observations or one-way ANOVA with a *post-hoc* test (Dunnett’s multiple comparison) for multiple group comparisons. Statistical significance was indicated by *p* < 0.05. Data are presented as mean ± SD. Each experiment was carried out in triplicate.

## Results

### Expression of COX-1 and COX-2 in Gastric Cancer Cells

**Figure 1:**
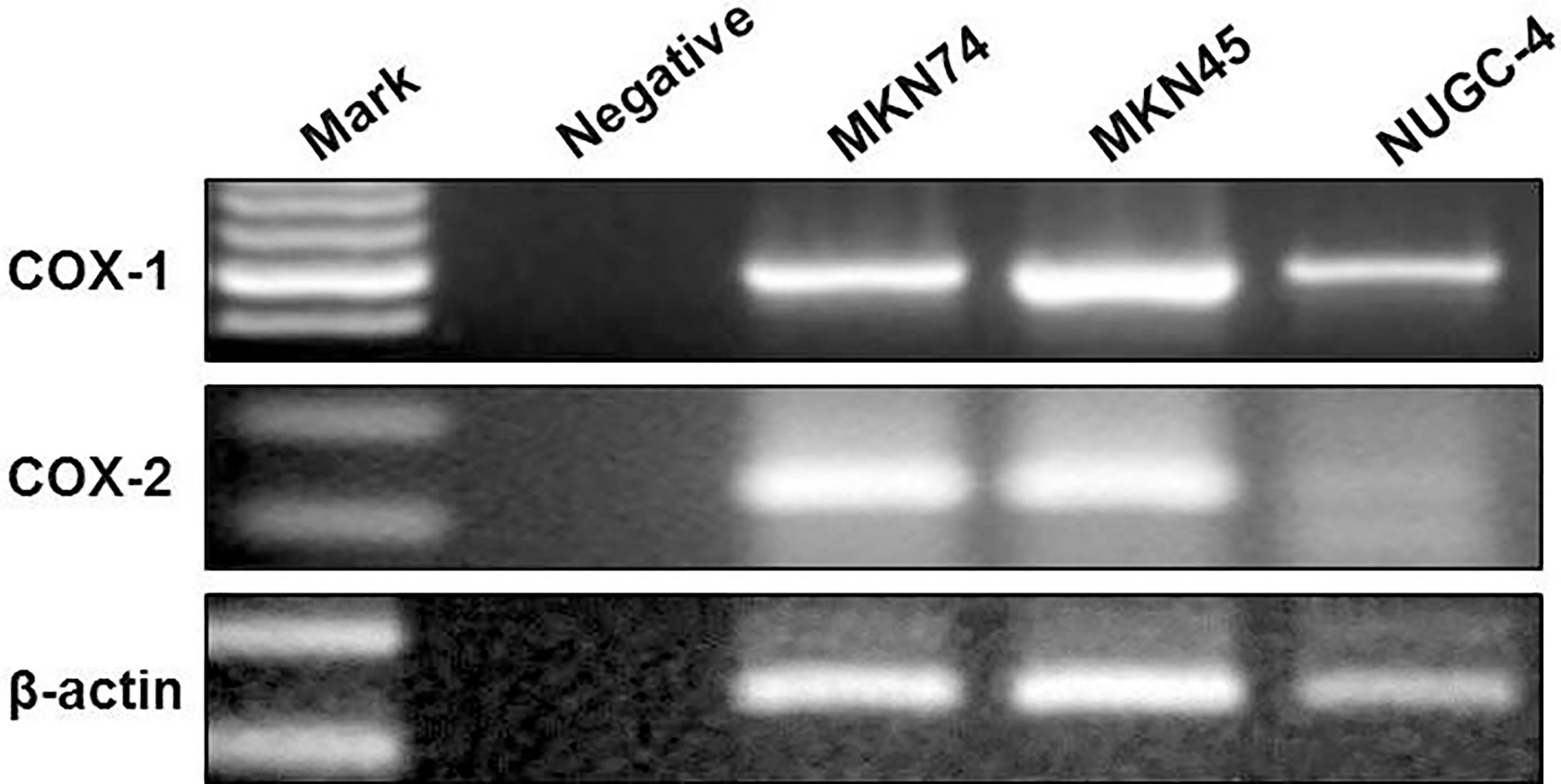
The expression of COX-1 and COX-2 in gastric cancer cells. COX-1 and COX-2 mRNA in gastric cancer cell lines were measured by RT-PCR. PCR products stained with ethidium bromide were displayed at 1.5% agarose gel electrophoresis. β-Actin served as a loading control. The experiments were performed at least thrice.

**Figure 2:**
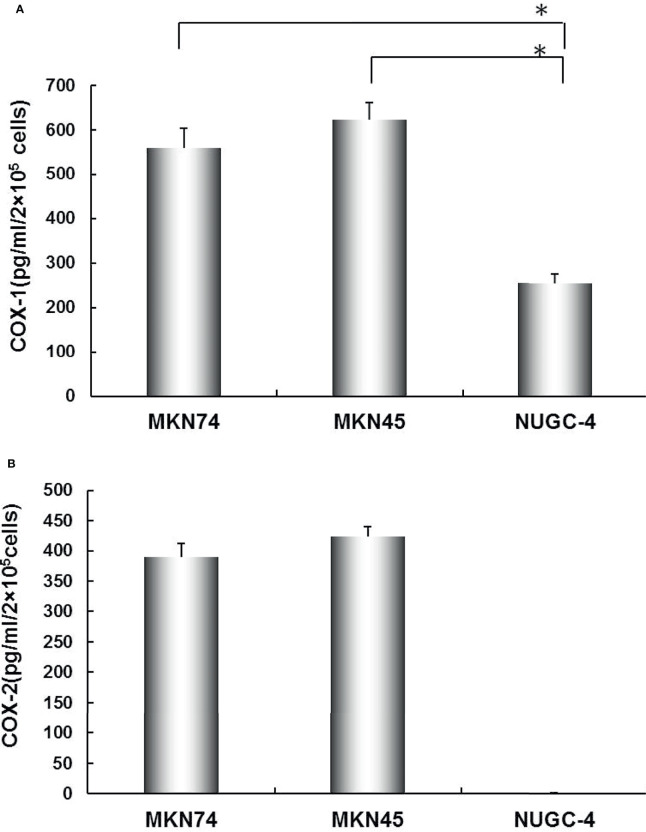
The secreted levels of COX-1 **(A)** and COX-2 **(B)** in gastric cancer cell lines. COX-1 and COX-2 protein concentration in MKN74, MKN45, and NUGC-4 cell culture medium was determined by ELISA. The values are expressed as mean ± SD. Multiple comparisons were analyzed by one-way ANOVA followed by Student-Newman-Keuls test; ^*^ *p* < 0.01. This experiment was carried out in triplicate.

COX-1 and COX-2 mRNA levels were determined in all gastric cancer cell lines by RT-PCR. The results showed that all gastric cancer cell lines were expressed as COX-1 mRNA. COX-2 mRNA was detected in MKN45 and MKN74 cells (**Figure 1**). Consistent with RT-PCR observations, COX-1 and COX-2 proteins secreted into cultured liquid supernatant were measured by ELISA. COX-2 proteins are 390.16 ± 22.19 and 423.05 ± 17.73 pg/ml/2 × 10^5^ cells in MKN74- and MKN45-cultured supernatant, respectively, but not detected in NUGC-4 cell-cultured supernatant. The secreted COX-1 protein was determined in the cultured liquid supernatant of MKN45, MKN74, and NUGC-4 cells. The secreted level of COX-1 in MKN74 (560.72 ± 43.09) and MKN45 (623.15 ± 38.59) is higher than NUGC-4 cells (25.93 ± 21.05). MKN74 and MKN45 are respectively compared with control, and *p*-value is less than 0.01 (**Figure 2**).

### Effect of COX-2 siRNA Transfection on Secretion of COX-2 Proteins in Gastric Cancer Cells

**Figure 3:**
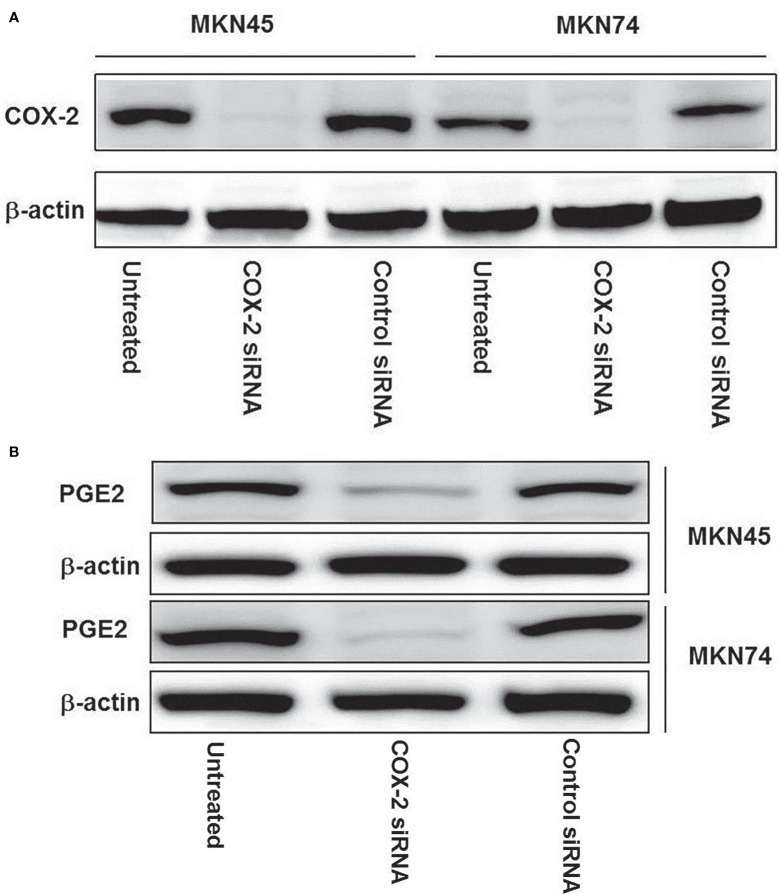
The expression of COX-2 protein in gastric cancer cell line after silencing of CXCL12 gene. Knockdown of COX-2 by COX-2 siRNA was confirmed by immunoblotting in expressed COX-2 gastric cancer cell lines: MKN74 and MKN45. COX-2 siRNA duplex oligoribonucleotides were transfected into cells for 48 h; the proteins were extracted and then subjected to Western blotting **(A)**. After being transfected with COX-2 siRNA in MKN45 and MKN74 cells, a significant inhibited expression of PGE2 was observed **(B)**. The experiments were performed in triplicate.

MKN45 and MKN74 gastric cells were transfected with siRNA, which specifically targets COX-2 genes; the expressions of COX-2 proteins were detected by immunoblotting. The results revealed that COX-2 gene silencing led to a near total loss of COX-2 expression, and compared with the untransfected and control siRNA groups and positive control β-actin, the expressions of COX-2 proteins in MKN45 and MKN74 cancer cells were significantly inhibited (**Figure 3A**). After transfecting MKN45 and MKN74 cells with COX-2 siRNA, significantly inhibited expressions of PGE2 were observed (**Figure 3B**).

### Effects of ω-3 PUFAs, ω-6 PUFAs, PGE2, and PGE3 on Gastric Cancer Cell Proliferation

**Figure 4:**
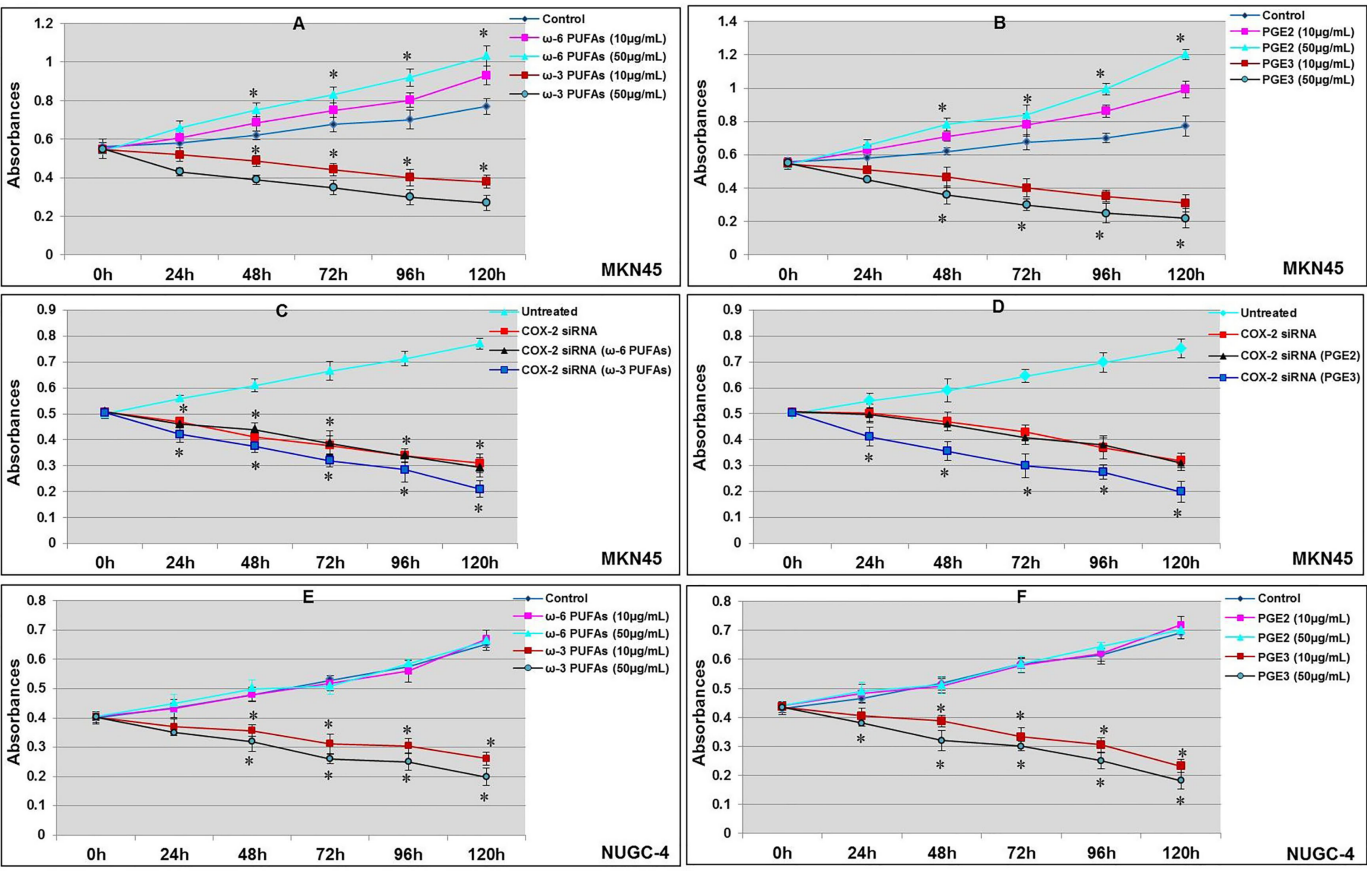
Effects of ω-3 PUFAs, ω-6 PUFAs, PGE2, and PGE3 on proliferation of gastric cancer cell. MKN45 gastric cancer cells incubated for 24 h; the proliferation of cancer cells was measured by WST-1 assay. The proliferation of MKN45 cells in ω-3 PUFA **(A)** and PGE3 **(B)** were significantly inhibited (compared with control groups, ^*^ *p* < 0.01); ω-6 PUFAs **(A)** and PGE2 **(B)** significantly promoted the proliferation (compared with control groups, ^*^ *p* < 0.01). MKN45 cells were transfected with COX-2 siRNA; the proliferation of MKN45 cells were significantly inhibited (compared with control group, ^*^ *p* < 0.01). PGE3 and ω-3 could also inhibit the proliferation of MKN45 (compared with COX-2 siRNA group, ^*^ *p* < 0.01). However, there were no significant changes in ω-6 and PGE2 **(C**, **D)**. The proliferation of NUGC-4 cells was significantly inhibited by the presence of ω-3 and PGE3. Moreover, there were no significant changes in the presence of ω-6 and PGE2 (compared with the control groups, respectively, ^*^ *p* < 0.01, **E**, **F**). Multiple comparisons used the method of one-way ANOVA and followed by the SNK test. Values are expressed as mean ± SD. Bars indicated SD, *p < 0.01. The experiments were performed at least thrice.

The gastric cancer cell line MKN45 can express COX-2. The cell proliferation curve showed that there was a difference in the proliferation of MKN45 gastric cancer cells following treatment with ω-6 and PGE2 after being cultured for 24 h; the proliferation of MKN45 cells in 0 and 50 μM of ω-6 and PGE3 group was significantly enhanced than those in the control groups after 48, 72, 96, and 120 h (^*^ *p* < 0.01, compared with the control groups); meanwhile, the proliferation of MKN45 cells was significantly inhibited by ω-3 and PGE3 in a concentration-dependent manner (compared with the control groups, respectively, ^*^ *p* < 0.01, as shown in **Figures 4A, B** **)**. After being transfected with COX-2 siRNA for 24 h, the proliferation of MKN45 cells was measured by WST-1 assay. The results showed that after COX-2 gene silencing, the proliferation of MKN45 cells was significantly inhibited (compared with the control groups, ^*^ *p* < 0.01). At the same time, ω-3 and PGE3 could have also inhibited the proliferation of MKN45, but in ω-6 and PGE2, nosignificant change was observed (compared with the COX-2 siRNA groups, ^*^ *p* < 0.01, as shown in **Figures 4C, D** **)**. The proliferation of NUGC-4 cells was significantly inhibited by the presence of ω-3 and PGE3. Moreover, there were no significant changes in the presence of ω-6 and PGE2 compared with the control groups, respectively (^*^ *p* < 0.01, **Figures 4E, F** **)**.

### Effects of ω-3 PUFAs, ω-6 PUFAs, PGE2, and PGE3 on Gastric Cancer Cell Invasion

**Figure 5:**
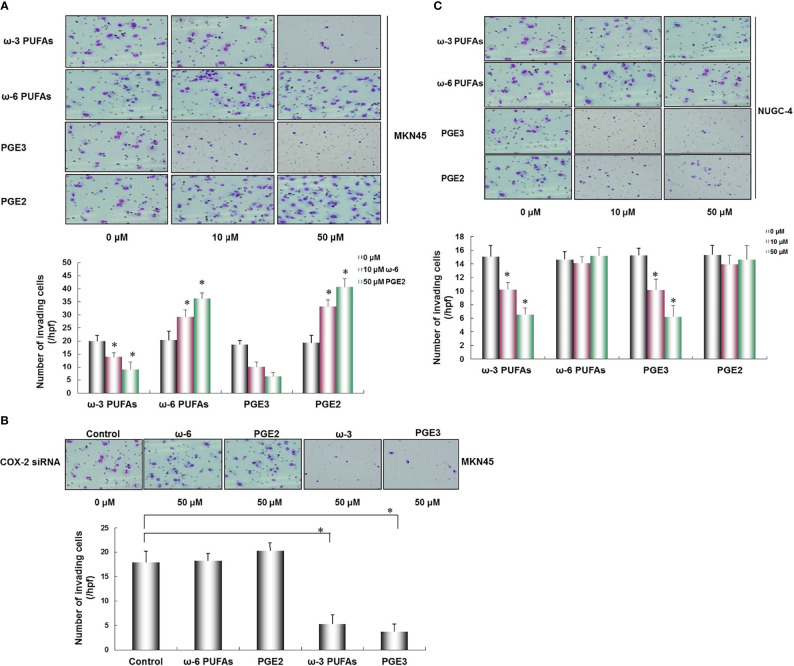
Effects of ω-3 PUFAs, ω-6 PUFAs, PGE2, and PGE3 on gastric cancer cell invasion. PGE3 and ω-3 inhibited the invasion of MKN45 cells, and as for ω-6 and PGE2, there was no significant effect on the invasive ability of MKN45 cells **(A)**. In the MKN45 cells transfected with COX-2 siRNA, no significant changes were found in the presence of ω-6 and PEG2 in MKN45 cells. PGE3 and ω-3 could significantly reduce the invasion capability of MKN45 cells compared with the control **(B)**. The invasion of NUGC-4 cells was inhibited by ω-3 and PGE2 in a concentration-dependent manner compared with the control (^*^ *p* < 0.01) and ω-6 and PGE2 cannot significantly influence the invasiveness of NUGC-4 cells **(C)**. Multiple comparisons used the method of one-way ANOVA followed by the SNK test. Columns, relative invading number. Bars indicate SD, ^*^ *p* < 0.01. The experiment was carried out in triplicate.

The results of transwell invasion assay showed that the COX-2-positive cell MKN45, ω-3, and PGE3 inhibited the invasion of MKN45 cells in a dose-dependent manner, and in ω-6 and PGE2, there was no significant effect on the invasive capability of MKN45 cells (^*^ *p* < 0.01, **Figure 5A**). After transfecting MKN45 cells with COX-2 siRNA, there were no significant changes in the presence of ω-6 and PGE2 in the invasive ability of MKN45. PGE3 and ω-3 could significantly reduce the invasion ability of MKN45 cells compared with the control (^*^ *p* < 0.01, as shown in **Figure 5B**). The invasion of gastric cancer cell NUGC-4 was inhibited by ω-3 and PGE2 in a concentration-dependent manner compared with the control (^*^ *p* < 0.01), and ω-6 and PGE2 cannot significantly influence the invasiveness of NUGC-4 cells (^*^ *p* < 0.01, as shown in **Figure 5C**).

### Effect of ω-3 PUFAs, ω-6 PUFAs, PGE2, and PGE3 on HUVEC Tube Formation

**Figure 6:**
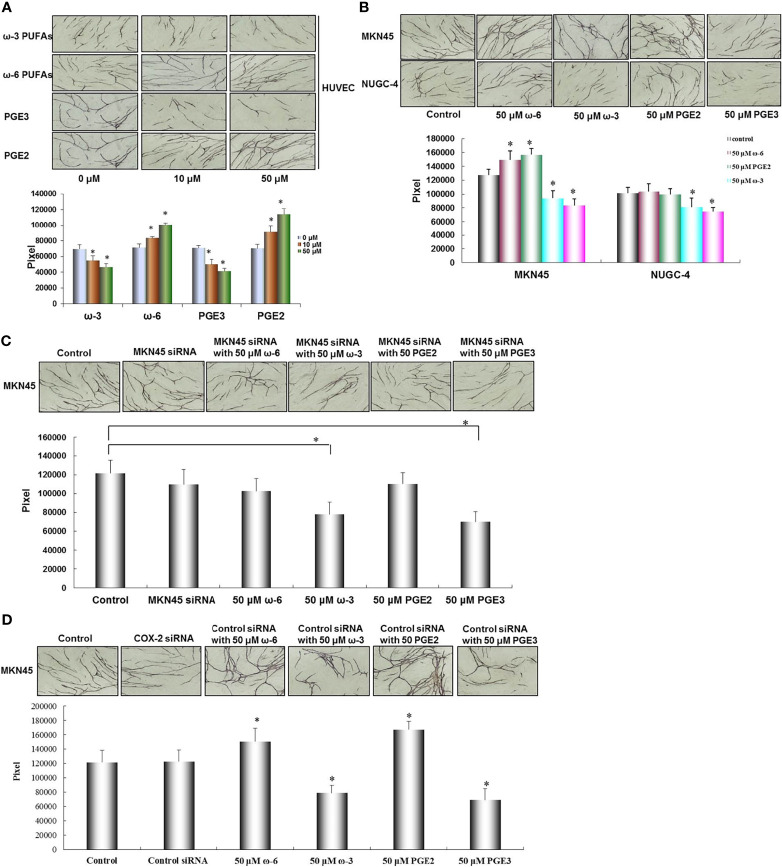
Effect of ω-3, ω-6 PUFAs, PGE2, PGE3, and gastric cancer cells on HUVEC tube formation. HUVEC tube formation was significantly promoted by ω-6 PUFAs and PGE2. On the contrary, HUVEC tube formation was also significantly inhibited by ω-3 PUFAs and PGE3 **(A)**. HUVEC tube formation was significantly enhanced by coculture with MKN45 cells compared with NUGC-4 cells. In MKN45 cocultured system, ω-6 PUFAs and PGE2 significantly promoted HUVEC tube formation, but this promoted action was inhibited by COX-2 siRNA. HUVEC tube formation was decreased by ω-6 PUFAs and PGE2 in MKN45 and NUGC-4 cocultured system **(B**, **C)**. To confirm the specificity of COX-2 siRNA for inhibition of COX-2 in MKN45 cells, control siRNA was set as a control in the MKN45 cells. The results showed that ω-6 and PGE2 significantly enhanced angiogenesis, while ω-3 and PGE3 significantly reduced tumor angiogenesis in cocultured system **(D)**. Multiple comparisons used the method of one-way ANOVA followed by the SNK test. Bars indicate SD, ^*^ *p* < 0.01. The experiments were performed in triplicate.

To measure the role of ω-3 PUFAs, ω-6 PUFAs, PGE2, and PGE3 in tube formation by HUVECs, we examined the effect of ω-3 PUFAs, ω-6 PUFAs, PGE2, and PGE3 on HUVEC tube formation using an angiogenesis assay. The HUVEC tube formation was significantly promoted in a dose-dependent manner following the presence of ω-6 PUFAs and PGE2 (compared with the control ^*^ *p* < 0.01). On the contrary, the HUVEC tube formation was also significantly inhibited by ω-3 PUFAs and PGE3 compared with the control (^*^ *p* *<* 0.01, **Figure 6A**).

### Effect of Gastric Cancer Cells and Presence of ω-3 PUFAs, ω-6 PUFAs, PGE2, and PGE3 on Tube Formation

In order to further pursue the effect of PUFA and its metabolites PGE2 and PGE3 on angiogenesis, focus should be made on the interaction between tumor cell and stromal cell by characterizing the angiogenic activity in cocultured system consisting of HUVECs, fibroblasts, and MKN45 or NUGC-4 gastric cancer cells. HUVEC tube formation was significantly enhanced by coculturing with MKN45 cells compared with NUGC-4 cells (^*^ *p* < 0.01). In MKN45 cocultured system, ω-6 PUFAs and PGE2 significantly promoted the HUVEC tube formation in a dose-dependent manner (compared with MKN45 only, ^*^ *p* < 0.01), but this promoted action was inhibited by COX-2 siRNA. Furthermore, the HUVEC tube formation was decreased by ω-6 PUFAs and PGE2 in MKN45 and NUGC-4 cocultured system (compared with the control, ^*^ *p* *<* 0.01, as shown in **Figures 6B, C** **)**. To confirm the specificity of COX-2 siRNA for inhibition of COX-2 in MKN45 cells, we set control siRNA in MKN45 cells as a control. The results showed that ω-6 and PGE2 significantly enhanced angiogenesis, while ω-3 and PGE3 significantly reduced tumor angiogenesis in cocultured system (**Figure 6D**).

## Discussion

The ω-3 and ω-6 polyunsaturated fats are the most common polyunsaturated fats. They play an indispensable role in maintaining the normal physiological metabolism of the human body and are essential fatty acids for the human body. ω-3 PUFAs mainly include alpha linolenic acid (ALA) and eicosapentaenoic acid (EPA); ω-6 PUFAs mainly include linoleic acid (LA) and arachidonic acid (AA). The conversion and utilization of ω-3 PUFAs and ω-6 PUFAs in the human body is a complex process. Cyclooxygenase can promote the conversion of AA and EPA into prostaglandin, thromboxane A (TXA), and other products (7). Recent studies have shown that ω-3 PUFAs have an inhibitory effect on the occurrence and progression of malignant tumors, while ω-6 PUFAs have a promoting effect; their mechanism of action may be related to the regulation of cyclooxygenase and prostaglandin synthetase (PGES), the main enzymes in the function and reaction of prostaglandin E3, and prostaglandin E2, and metabolites of ω-3 PUFAs and ω-6 PUFAs (17, 18). ω-6 PUFAs bind to COX-2 in the human body to generate PGE2, while PGE2 can induce cell proliferation and stimulate the expression of BLC-2 protein (BLC-2 protein inhibits apoptosis) to imbalance cell proliferation and apoptosis and promote the occurrence of tumors; PGE2 can also promote extracellular matrix degradation and produce thromboxane to promote platelet aggregation, which is conducive to the invasion and metastasis of cancer cells. While ω-3 PUFAs produce PGE3 after binding to COX-1, PGE3 can inhibit the production of PGE2 and can inhibit phospholipase A2 (PLA2), phosphatidylinositol-specific phospholipase C (PI-PLC), nuclear factor-κB, and COX-2 activities, which in turn reduce the proliferation and invasion of tumor cells and play a role in inhibiting the growth and metastasis of malignant tumors (8, 12, 19–21). Previously, we retrospectively analyzed the clinical data of 115 patients with radical resection of colorectal cancer and found that the positive rate of prostaglandin E2 expression in colorectal cancer tissues was 87.8%, which was significantly higher than that in normal colorectal mucosal tissues and correlated with the depth of invasion and lymph node and liver metastasis of colorectal cancer; it was positively correlated with the expression of cyclooxygenase 2; the 5-year cumulative survival rate was 63.6% in patients with double-negative PGE2 and COX-2 and 37.8% in patients with double-positive expression (22). Thus, PGE2 and COX-2 downstream of ω-6 PUFAs can be used as important markers for the clinical evaluation of metastasis of colorectal cancer and are important for patient prognosis assessment.

ω-6 PUFAs rely on the catalytic effect of COX-2 to generate PGE2 in the body, which can stimulate the expression of Bcl-2 protein to imbalance cell proliferation and apoptosis and thus promote tumor progression. PGE2 can also enhance the degradation of extracellular matrix, which further promotes the invasion and metastasis of cancer cells (18). ω-6 PUFAs in the microenvironment can upregulate PGE2 production in colorectal cancer cells and promote the transformation of myeloid-inhibiting cells (MDSC) into M2 macrophages (23); hypoxia-inducible factor-1α (HIF-1α) secreted by M2 macrophages promotes tumor invasion and metastasis by inducing the expression of COX-2 and PGE2 in stromal cells and tumor cells in the hypoxic microenvironment (24). HIF-1α derived from M2 macrophages elevates the secretion of CXCR4 in cancer cells to promote colorectal liver metastasis (25). While ω-3 PUFAs produce prostaglandin E3 (PGE3) in response to COX-1, PGE3 inhibits the proliferation and invasion of tumor cells by downregulating the expression of phospholipase A2 phosphatidylinositol-specific phospholipase C (PI-PLC), nuclear factor (NF-κB), and COX-2. PGE3 can also inhibit the metastasis of colorectal cancer by downregulating colorectal cell adhesion factors and the formation of new blood vessels, and ω-3 PUFAs upstream of PGE3 have a potential application value in the treatment of colorectal cancer as a target of antitumor angiogenesis (26, 27).

On the basis of previous studies, this experiment focused on exploring the role of ω-3, ω-6, PGE2, and PGE3 in gastric cancer metastasis, and the results showed that the expression of PGE2 and COX-2 in gastric cancer cell lines was closely related to their liver metastasis, that is, PGE2 and COX-2 were expressed in cell lines MKN45 and MKN74 with high liver metastasis, while PGE3 and COX-1 were expressed in cell lines with high and low liver metastases. The ω-6 PUFAs in the tumor microenvironment is converted into PGE2 that promotes tumor growth by binding to COX-2 in gastric cancer cells, and PGE2 can progressively enhance the proliferation, invasion, and angiogenesis of gastric cancer cells over the increase of concentration. On the one hand, ω-3 PUFAs can inhibit the activity of COX-2 and reduce the production of PGE2, thereby inhibiting the proliferation, invasion, and angiogenesis of tumor cells; on the other hand, ω-3 PUFAs can compete with ω-6 PUFAs to bind to COX-1 to produce PGE3, which can significantly inhibit the proliferation, invasion, and angiogenesis of tumor cells. In addition, after silencing COX-2 gene, ω-6 inhibits the proliferation, invasion, and angiogenesis of gastric cancer cells. In order to detect the effect of unsaturated fatty acids in tumor microenvironment on gastric cancer angiogenesis, we used gastric cancer cells and stromal cells to construct a coculture system to culture angiogenesis *in vitro* and detected the effect of gastric cancer cells with a different expression of COX-2 on angiogenesis. The effect of MKN45 on the angiogenesis of HUVEC in COX-2-positive gastric cancer cells was significantly stronger than that in COX-2-negative gastric cancer cells (NUGC-4); ω-6 PUFAs could promote the angiogenesis of COX-2-positive gastric cancer cells, while ω-3 PUFAs could inhibit the angiogenesis of COX-1-positive gastric cancer cells. The above results demonstrated that the effect of ω-3 and ω-6 PUFAs on gastric cancer metastasis was mainly achieved by regulating the physiological functions of COX and PGE. ω-6 enhances the metastatic potential energy of gastric cancer cells by being converted into PGE2 that promotes tumor growth after binding to COX-2; ω-3 can inhibit the activity of COX-2 and reduce the production of PGE2 on the one hand, thereby inhibiting the metastatic potential energy of gastric cancer; on the other hand, ω-3 can compete with ω-6 to bind to COX-1 to produce PGE3 so as to inhibit the metastatic potential energy of gastric cancer. Taking PUFAs and its intermediate metabolites as interference factors, the *in vitro* simulation experiment and exploration experiment of tumor internal environment using a coculture system can more objectively and truly reproduce and observe the effect of PUFAs on the microenvironment of gastric cancer cells, which plays an irreplaceable important role in understanding the specific growth, invasion, and metastasis mechanism of tumor cells, and also plays an important theoretical foundation for the next *in vivo* experiment and clinical trials.

At present, there are a few studies on ω-3 PUFAs, ω-6 PUFAs, PGE2, and PGE3 in gastric cancer, and the mechanism and clinical significance of the effect of polyunsaturated fatty acids on the occurrence, development, and metastasis of gastric cancer remain to be more deeply and comprehensively studied. Through further *in vitro* and *in vivo* experiments at a later stage, our team will find a suitable ratio of two fatty acids or a suitable concentration of COX-2 inhibitor, in order to inhibit the invasion and metastasis of tumor cells, finally providing a new way for clinical prevention and treatment of gastric cancer.

## Data Availability Statement

The original contributions presented in the study are included in the article/supplementary material. Further inquiries can be directed to the corresponding author.

## Author Contributions

JM, CZ, and WL designed the project. JM and CZ wrote the manuscript. LL, JD, and CP finished **Figures 1**, **2**. BC finished **Figure 3**. YC finished **Figure 4**. JM and YW finished **Figures 5**, **6**. All authors reviewed the manuscript. All authors listed have made a substantial, direct, and intellectual contribution to the work and approved it for publication.

## Funding

This work was supported by Grants from the Natural and Science Foundation of China (Grant No. 81260325), Key Natural Science Research Projects in Universities of Anhui Province (Grant No. KJ2019A0396), and Bengbu Medical College Gastric Cancer Multidisciplinary Diagnosis and Treatment Innovation Team Project (Grant No. BYKC 201907).

## Conflict of Interest

The authors declare that the research was conducted in the absence of any commercial or financial relationships that could be construed as a potential conflict of interest.

## Publisher’s Note

All claims expressed in this article are solely those of the authors and do not necessarily represent those of their affiliated organizations, or those of the publisher, the editors and the reviewers. Any product that may be evaluated in this article, or claim that may be made by its manufacturer, is not guaranteed or endorsed by the publisher.
